# Fingerprint Liveness Detection in the Presence of Capable Intruders

**DOI:** 10.3390/s150614615

**Published:** 2015-06-19

**Authors:** Ana F. Sequeira, Jaime S. Cardoso

**Affiliations:** 1 INESC TEC—INESC Technology and Science, Campus da FEUP, Rua Dr. Roberto Frias, Porto 4200-465, Portugal; E-Mail: jsc@inescporto.pt; 2 Departamento de Engenharia Eletrotécnica e de Computadores, Faculdade de Engenharia da Universidade do Porto, Rua Dr. Roberto Frias, Porto 4200-465, Portugal

**Keywords:** biometrics, liveness detection, fingerprint, supervised classification, semi-supervised classification

## Abstract

Fingerprint liveness detection methods have been developed as an attempt to overcome the vulnerability of fingerprint biometric systems to spoofing attacks. Traditional approaches have been quite optimistic about the behavior of the intruder assuming the use of a previously known material. This assumption has led to the use of supervised techniques to estimate the performance of the methods, using both live and spoof samples to train the predictive models and evaluate each type of fake samples individually. Additionally, the background was often included in the sample representation, completely distorting the decision process. Therefore, we propose that an automatic segmentation step should be performed to isolate the fingerprint from the background and truly decide on the liveness of the fingerprint and not on the characteristics of the background. Also, we argue that one cannot aim to model the fake samples completely since the material used by the intruder is unknown beforehand. We approach the design by modeling the distribution of the live samples and predicting as fake the samples very unlikely according to that model. Our experiments compare the performance of the supervised approaches with the semi-supervised ones that rely solely on the live samples. The results obtained differ from the ones obtained by the more standard approaches which reinforces our conviction that the results in the literature are misleadingly estimating the true vulnerability of the biometric system.

## 1. Introduction

Biometric recognition is nowadays a mature technology used in many government and civilian applications such as e-passports, ID cards, and border control. Examples include the US-Visit (United States Visitor and Immigrant Status Indicator Technology) fingerprint system, the Privium iris system at Schiphol airport, and the SmartGate face system at Sydney Airport [1]. Recognition systems based on fingerprints (FRS) in particular are widely used, in fact, probably this was the first biometric trait to be used to identify people.

Fingerprints are small lines/ridges and valleys in the skin of fingertips. Their configuration is formed at around the seventh month of fetus development due to a combination of genes and environmental factors and do not change throughout life (except if an accident, such as a severe burn, happens) [2,3]. The influence of environmental factors in the fingerprint formation results in such variations in its configuration that it is considered impossible to have two fingerprint looking exactly alike [2,4].

**Figure 1:**
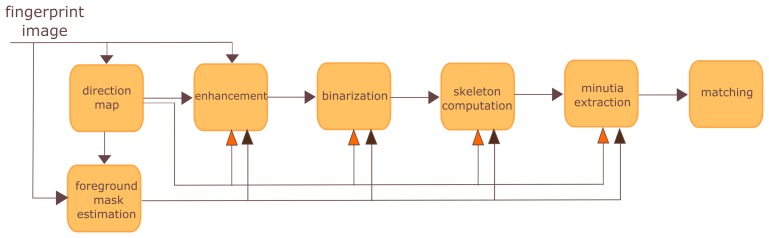
Fingerprint recognition system block diagram.

Most of the fingerprint recognition and classification algorithms perform some preprocessing, segmentation and enhancement steps to simplify the task of minutiae extraction. The main steps of a FRS system may be seen in Figure 1. It is worth highlighting the estimation of the foreground mask which is a crucial step to all the following stages of the process.

**Figure 2:**
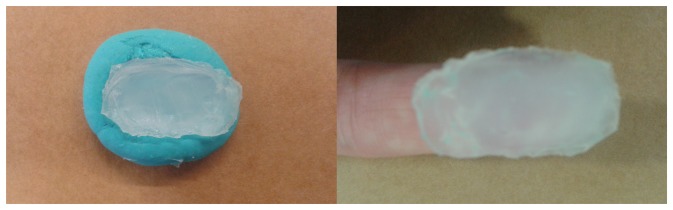
Finger Play-Doh mold and silicon model.

Biometric recognition systems in general, and FRS in particular, can be spoofed by presenting fake or altered samples of the biometric trait to the sensor used in a specific system. Liveness detection techniques and alteration detection methods are both methods included in the *presentation attack detection* (PAD) methods [5]. Concerning spoofing attacks with fake samples, the fake samples can be acquired with or without user cooperation: an authorized user may help an hacker to create a clone of his fingerprint; or the fingerprint may be obtained from a glass or other surface (latent fingerprints) [2,6]. Latent fingerprints can be painted with a dye or powder and then “lifted” with tape or glue. However, these prints have, usually, low quality as they can be incomplete or smudged and thus are not very accurate. The easiest way of creating a fake sample is by printing the fingerprint image into a transparent paper. A more successful method is to create a 3D fake model with the fingerprint stamped on it. This can be done by creating a mold that is then filled with a substance (silicon, gelatin, Play-Doh, wax, glue, plastic). Then this mold is used to create a thick or thin mold that an intruder can use, as depicted in Figure 2.

Liveness detection methods can be categorized as hardware or software-based whether the detection is performed through additional hardware or by processing the obtained image [7]. Hardware-based solutions work by measuring some physical characteristics (such as blood pressure, temperature, pulse, or pupil dilation, voluntary eye blink, among others) and have the disadvantage of being expensive and requiring intervention at the device level. Memon *et al.* [8] make a detailed classification of the several methods of hardware based fingerprint liveness detection methods dividing them in three categories: *biological or involuntary signals, skin physiology characteristics* and *stimulation response*. Software-based liveness detection methods can be divided in two categories based on whether they work with a single static 2D scan (static methods), or need 2D scans at different time points during the acquisition process that support the observation of the dynamic properties (dynamic methods) [5]. The static methods comprise the analysis of textural features; sweat pores; ridgey and valley structure; perspiration; and surface coarseness [5]. We confined this work to software-based approaches and within these to static features, in particular texture based statistics.

This paper is organized as follows. In Section 2 some approaches found in literature are presented. In Section 3, we point out some methodological limitations of the current research and present the proposed approach and its motivation. In Section 4, the dataset of images is presented in Section 4.1, the segmentation method is presented in Section 4.2, the feature extraction methods are presented in Section 4.3, the general information on the classifiers is presented in Section 4.4, the classification studies are presented in Sections 4.5, 4.6.1 and 4.6.2 and finally the metrics used for the evaluation are presented in Section 4.7. The results and their discussion are presented in Section 5. Finally, in Section 6 we draw some conclusions from the results obtained and point the direction of future works.

## 2. Related Work

Nikam and Agarwal proposed several methods based on the use of statistical analysis of the fingerprint scans, particularly, they propose the use of Local Binary Patterns (LBP) along with the wavelet transform [9]. It is known that an LBP's histogram can be a powerful texture feature and thus can be used to determine whether a fingerprint is real or fake. The same authors published several works proposing the use of Gray Level Co-occurrence Matrices (GLCMs) combined with diversified methods such as Gabor filters [10], wavelet transform [11] and curvelet transform [12]. In a recent work, Nogueira *et al.* [13] propose two different methods, one performing the feature extraction with LBP and another using Convolutional Networks (CN) for the first time in this task. The methods perform dimension reduction using Principal Component Analysis (PCA) and use a Support Vector Machines (SVM) with Radial Basis Function (RBF) kernel to perform classification. Ghiani *et al.* have proposed a method for liveness detection by using Local Phase Quantization (LPQ) [14]. The LPQ is a blur insensitive texture classification method. As it is able to represent all spectrum characteristics of images in a compact feature representation, avoiding redundant or blurred information, the authors believe that it could be used in this field. They used the four data sets collected for the Second International Fingerprint Liveness Detection Competition (LivDet11) [15] to test the algorithm. Ghiani *et al.* [16] also proposed the use of Binarized Statistical Image Features (BSIF) to detect the vitality of fingerprints. This approach has already been tested for face recognition and texture classification. Their goal is to obtain statistically meaningful representation of the fingerprint data by learning a fixed set of filters from a small set of images. They also claim that through learning, it is possible to adjust the descriptor length to the unusual characteristics of a fingerprint. Ghiani *et al.* tested this algorithm with the four LivDet2011 [15] datasets, obtaining promising results. However, there are still some problems with this algorithm, such as finding the right window size or the length of the binary string that results from the application of the filters to the image. In these two latter works, the classifier used was a linear support vector machine. Gragnaniello *et al.* [17] investigate the use of a local discriminatory feature space, called Weber Local Descriptor (WLD) for fingerprint liveness detection. This descriptor consists of two blocks: differential excitation and orientation. These are then evaluated for each pixel of the image and concatenated into an histogram that is used to build the discriminatory features. A linear support vector machine classifier is then used to classify the images. These authors have tested this method with both LivDet2009 [18] and LivDet2011 [15] datasets and propose the combination of this method with the LPQ [14] in order to obtain better results. Galbally *et al.* [19] use quality related features in their liveness detection work. The extracted features are ridge strength, ridge continuity and ridge clarity. They claim that those features can be extracted from the following quality measures: local angle, power spectrum and pixel intensity. The classification step is performed using linear discriminant analysis. This study presented an overall rate of 90% correctly classified samples, tested on a challenging database comprising over 10,500 real and fake images. This large database is created from the images of LivDet2009 [18] and ATVS [20] databases. Tan and Schuckers [21] proposed a new method for fingerprint liveness detection based on ridge signal analysis and valley noise analysis. They aim to quantify perspiration patterns along ridge in live samples and quantify noise patterns along valleys in fake samples. They present the performance of several standard pattern recognition classifiers including SVM. Their results show that the performance can reach 99.1% of correctly classified images. They have also proposed another method based on the statistics of Wavelet signal processing [22] aiming to detect the perspiration phenomenon using only a single image. In this work, the authors use classification trees for the classification task. Manivanan *et al.* [23] proposed a method to detect pores as a sign to fingerprint vitality using only one fingerprint image then applying two filtering techniques: highpass and correlation. The main reason of using highpass filter was to extract active sweat pore, then a correlation filter was used to locate the position of pores. Recently, Johnson and Shuckers [24] proposed a pore analysis method which still classifies the pores using their perspiration activity even if they are well represented in high quality fake fingers. A new invariant descriptor of fingerprint ridge texture, histograms of invariant gradients (HIG), is proposed by Gottschlich *et al.* [25] and again a SVM is used for classification. Warwante *et al.* [26] studied how the wavelet transform can be applied to fingerprint liveness detection. In this work, it is stated that wavelet analysis can help minimizing the effect of ridge and valley pattern when estimating the surface coarseness because it allows the study of the input image at different scales. They have created a high resolution database to which they then applied the proposed algorithm. Although they obtained positive results, one cannot say that the same would occur with images with less quality. In a recent work, Menotti *et al.* [27] proposed two general-purpose approaches to build image-based anti-spoofing systems with convolutional networks for several attack types in three biometric modalities, namely iris, face, and fingerprint. One technique is hyperparameter optimization of network architectures and the second lies at the core of convolutional networks and consists of learning filter weights via the well-known back-propagation algorithm.In this latter work the authors note that most of the state-of-the-art methods rely on hard-coded features sometimes exploring quality metrics directly related to the biometric trait that is being treated, such as directionality and ridge strength; or general texture patterns such as LBP- or LPQ-based methods. In an innovative approach, their work is inspired by the recent success of *Deep Learning* techniques in several vision tasks. Some previous works that explore filter learning through natural image statistics, such as BSIF [16], or another that uses CN [13], are examples of this new research trend, which seeks to model the problem by learning features directly from the data. One basilar idea of this approach is to assume little *a priori* knowledge about acquisition-level biometric spoofing and explore deep representations of the data.

## 3. Methodological Limitations of Current Research and Proposed Approach

**Figure 3:**
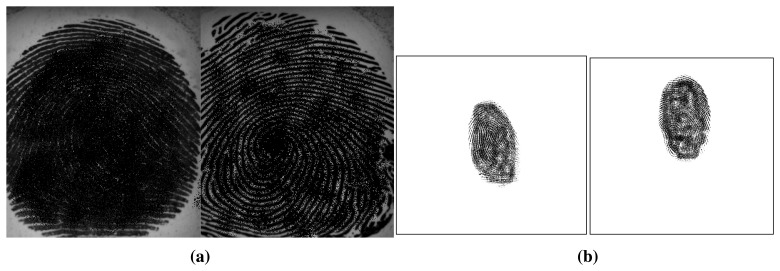
Example of differences in background area in two pairs of real and fake fingerprint images from two different datasets (images were degraded for privacy purposes). (**a**) Pair of real and fake fingerprint images with a small area of background (Biometrika dataset); (**b**) Pair of real and fake fingerprint images with a significant area of background (Crossmatch dataset).

One limitation of several of the existing procedures is the inclusion of the background in the liveness decision. Center your attention in Figure 3a,b. In both cases the left fingerprint is real and the right is fake. However, in the first pair we may observe that the fingerprint occupies the most part of the image and, on the other hand, in the second pair, the background represents a significant area of the image and naturally it will dominate the representation (LPB, GLCMs, *etc.*). In the first pair it is expected that, for a good set of discriminant liveness detection features, the image features differ significantly between the real and the fake samples. In the second pair, as the background is very similar and occupies a considerable part of the image, the liveness system is likely to find much more challenging to discriminate the real from the fake in this second case than in the first case. Therefore, the influence of background in the decision making is not enabling the system to capture the true vulnerability of the biometric system and the security level may be underestimated in this case. Additionally, we are not interested in assessing the liveness of the background (which should always be lifeless) but only in assessing the liveness of the fingerprint. It is therefore with surprise that one verifies that fingerprint foreground segmentation is well established in the recognition works and often forgotten in the liveness detection works. We found in fingerprint liveness detection literature examples of inclusion of background and examples of manual or random crops to obtain the fingerprint partially [21,26] but rarely an automatic segmentation step included in the process [19]. This observation shows how the segmentation step has been disregarded in most of the liveness detection methods.

**Figure 4:**
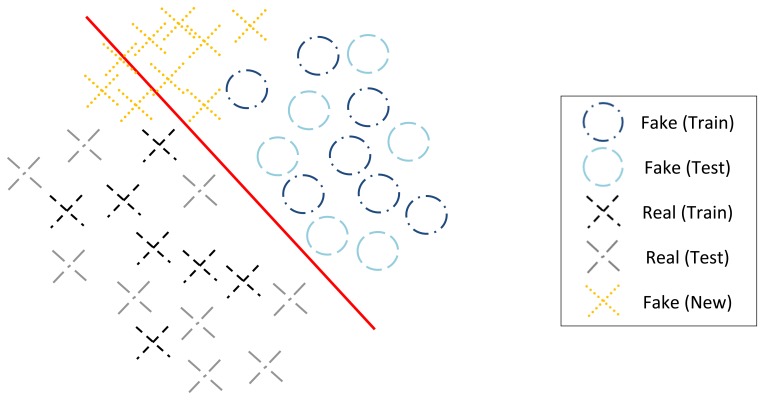
Illustrative Example. Black crosses and dark blue circles are fake and real samples in the training set. Light blue circles are real samples; gray crosses are fake samples from materials present in the training set; orange crosses are fake samples from a new material. The red curve represents the model learnt from the training samples.

Another methodological limitation is that models are designed and evaluated using fake samples of one type of material individually. The systems are developed and tested under the assumption that the intruder will fabricate the fake fingerprint by employing one of the materials used for training, resulting in optimistically estimating the security level of the system. At the design time, the developer assumes to possess labeled data representative of the real and fake samples and therefore resorts to standard binary classifiers (which may follow generative principles, like Naive Bayes, or be non-probabilistic, like SVM). The binary classifiers adopted to make the decision between real and fake samples implicitly assume that the training samples are representative of the complete population, with the test data to which the system is applied coming from the same distribution as the training data. Although that is a fair assumption for the real samples, it may be a crude model for fake samples created from a new material, see Figure 4. It may well happen that there is a mismatch between the distribution of the observations in training and testing data. In recent years, machine-learning researchers have investigated methods to handle mismatch between the training and test domains, with the goal of building a classifier using the labeled data in the old domain that will perform well on the test data in the new domain [28]. Recent works already depart from this conventional approach. Marasco and Sansone [29] performed an experimental comparison among fingerprint liveness detection approaches adopting materials for training different than those adopted for testing and concluding that the performance significantly

decreases in this conditions. Although standard binary classifiers were used, this work encourages the adoption of different classification approaches in the liveness detection context. Another approach to the problem was presented by Rattani and Ross [30], consisting on designing an on-line scheme for automatic adaptation of a liveness detector to novel spoof materials encountered during the operational phase, which can lead to significant improvements in the performance. This approach is likely to accommodate well small differences (drifts) between materials, but it will likely under-perform when the new material is significantly different from the ones seen before. Still, this approach is in a sense complementary to ours.

### 3.1. Proposed Approach

A first methodological contribution is the inclusion of an automatic fingerprint segmentation step before feature extraction for liveness detection. Instead of extracting features from all the acquired image, the image is segmented to find the mask defining the fingerprint region. The features can then be computed based solely on the information inside the mask (or the bounding box). This way of proceeding should lead to more realistic estimates of the vulnerability of the systems.

As new materials for fraudulent spoof attacks are going to continuously appear and become more and more sophisticated it is our conviction that the path to pursue is in the direction of using less and less information of spoofing materials and rely more strongly on the live samples. Our second methodological contribution includes: (a) the realistic estimation of the performance in the presence of new materials used to fabricate the fake samples; (b) the use of decision models that rely only on the information from the real samples to detect the liveness.

The traditional approaches to liveness detection in general may be included in the category of *supervised detection* considering that they assume the availability of a training data set which has labeled instances for real and fake classes. Any unseen data instance is compared against the model to determine which class it belongs to [31]. Having in mind that in real-world solutions the system is not “aware” of the kind of attack that might be performed, in this work we explore other approaches in which we do not assume knowledge about the fake samples used in a spoofing attack. This study can be framed in a *semi-supervised detection* category considering that we use the real/live samples to train our model but not the fake/spoof ones. The techniques that operate in a semi-supervised mode assume that the training data has labeled instances for only the live class. Since they do not require labels for the fake class, they are more widely applicable than supervised techniques and do not overfit to the materials in the training set.

An innovative approach in the iris liveness detection context (concerning spoofing attacks using contact lenses) was presented by Bowyer and Doyle [32]. In this work, the authors compare a baseline experiment where the training and test datasets each contained iris images with three lens types. Any of several classifiers could be trained with local binary pattern texture features and achieve 100 percent correct classification of the test set. Then they performed another experiment using the same texture features, classifiers, and images, but the training set contained iris images with two of the three lens types and the test set contained iris images with the third lens type. The results obtained varied according to combinations tested but for the lower results the classification accuracy was no better than 75%. The authors concluded, that these results illustrate how experimental results obtained using the same lens types in both the training and testing data can give a very misleading idea of the accuracy that will result when a new lens type is encountered. Although this approach still fits in the supervised study (considering that in training both live and spoof samples are used) it goes further than the most traditional approaches since in the test step a new type of spoof samples is presented. We hint that the kind of conclusion achieved (in the referred work by Bowyer and Doyle) will hold regarding other biometric traits and particularly in the fingerprint liveness study. Therefore, the path to pursue will put us more distant from the supervised classification approach.

## 4. Experimental Methodology

### 4.1. Datasets

The experiments were run in the fingerprint datasets made available for the LivDet2013 [33] competition. The images of these datasets were acquired with four different devices: Biometrika, Crossmatch, Italdata and Swipe. All this four sensors acquire the fingerprint images by contact of the finger with the sensor, however, the first three may be classified as *touch* whilst the fourth is classified as *swipe* due to the fact that this latter requires the subjects to swipe their finger over the sensor surface [5]. More than 4000 images were taken with each of the aforementioned devices. In order to build the fake part of the database, seven different materials were used: Ecoflex (1), Wood Glue (2), Latex (3), Modasil (4), Gelatin (5), Body Double (6) and Play-Doh (7). The fake images come from approximately 100 fingers of 20 people for the Crossmatch and Swipe datasets and from 100 fingers of 15 people for the Biometrika and Italdata datasets. Also, for the Crossmatch and Swipe datasets cooperative methods were used and for the other two the fingerprints were acquired through non-cooperative ways. The living images come from 440 fingers of 44 people for the Crossmatch dataset, from 250 fingers of 50 subjects for Swipe and from 300 fingers of 30 subjects for Biometrika and Italdata datasets. In Table 1 summarized the most relevant characteristics of the images obtained with each sensor.

### 4.2. Segmentation Method

**Figure 5:**
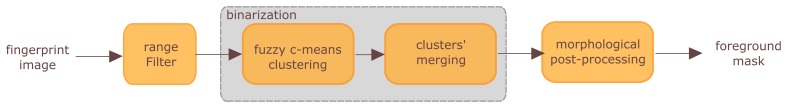
Main steps of the segmentation method (adapted from [34]).

The segmentation of the fingerprints was performed using the method presented by Ferreira *et al.* [34]. The method follows the morphological fingerprint segmentation algorithm presented in [35] suggesting a more robust binarization method instead of the simple adaptative thresholding binarization used. In this method, first, a block-wise range filter is applied to the grey-scale fingerprint image in order to enhance the ridges. Afterwards, the resulting range image is binarized using the Fuzzy c-means (FCM) algorithm along with a clusters' merging procedure. Finally, a set of morphological operations is applied to the binary image in order to compute the final foreground mask. The high-level operations that compose the algorithm are presented in Figure 5.

**Figure 6:**
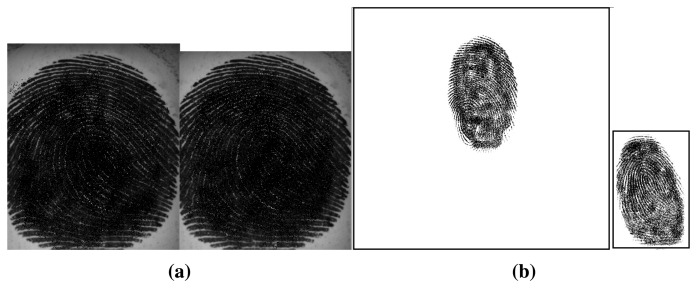
Examples of the result obtained by the segmentation method (images were degraded for privacy purposes). (**a**) Pair of original and segmented fingerprint images (Biometrika dataset); (**b**) Pair of original and segmented fingerprint images (Crossmatch dataset).

In our work we used the region of the fingerprint comprised by the bounding box obtained after the foreground mask computation. In the case of fingerprints images where the fingerprint occupied the most part of the image, see Figure 6a, the segmentation will not impact so much the feature extraction step. However, when the background area is significant in the fingerprint image, see Figure 6b, we expect that the segmentation step will lead to an improvement in the discriminant capacity of the feature extraction.

### 4.3. Feature Extraction

The feature extraction was performed using two state-of-the-art feature extraction methods, initially proposed for iris liveness detection. For fingerprint, as for iris, the texture is a significant feature making texture features good descriptors for both.

#### 4.3.1. Weighted Local Binary Patterns (wLBP)

This method [36] combines Local Binary Patterns (LBP) [37,38] with a Scale Invariant Feature Transform (SIFT) [39]. The process starts with the generation of a Gaussian scale space. The output of this operation is a smoothed image in six scales. For each scale, the gradient orientation of each pixel is calculated and an histogram of gradient orientations is created. Every histogram is then converted into a descending rank, from 7 to 0. The LBP method labels the pixels of an image by comparing them with their neighborhood. Combining the LBP method with SIFT results in weighted maps. Three simple maps are constructed using the 3 first scales and a fourth map results from the combination of the last scales. Each map is divided in 8 by 8 blocks and three statistical features are extracted from each block (standard deviation of the wLBP histogram, mean of wLBP map and standard deviation of the wLBP map). That results in a 768 dimensional feature. The only adaptation made in our implementation is the use of all the 8 × 8 blocks instead of discarding the first and last rows of the image as done in the original method to avoid noisy regions caused by eyelid occlusions. For more details on the method see the work of Zhang *et al.* [36]. The implementation we used was partially implemented in house and partially a third party implementation. For the SIFT part of the method we used the VLFeat Open Source implementation [40] (version *V LFeat*0.9.20 for Windows). All the parameters of the SIFT method are defined by the implementation and details can be obtained in the documentation. The remaining part of the method was implemented in Matlab (Mathworks version R2012b, The MathWorks, Inc., Natick, MA, USA, 2012).

#### 4.3.2. Gray Level Co-Occurence Matrices (GLCM)

This method [41] is based on GLCM [42] which characterize the relationship between neighboring pixels. Fourteen features are extracted from each GLCM matrix: angular second moment, contrast, correlation, variance, inverse difference moment, sum average, sum variance, sum entropy, entropy, difference variance, difference entropy, two information measures of correlation and the maximal correlation coefficient. These features are orientation dependent so four values can be obtained for each feature based on the four orientations (0°, 45°, 90°, 135°). The mean and standard deviation of the four values (four orientations) of each 14 measures, compose a set of 28 features. For more details on the method see the work of Wei *et al.* [41]. The method was implemented in Matlab (Mathworks, version R2012b).

### 4.4. Classifiers and Parameter Optimization

Our experiments comprise different classification approaches. We start with the traditional approach using a binary classifier and training and testing within each dataset, then we introduce modifications in the training and testing datasets and end up with a study where only the information about the live samples is used for training our models. For the classification task we used SVM [43], one-class SVM (OCSVM) [44,45] and a Mixture of Gaussians Model (GMM).

In the first three studies (1, 2 and 3.1), we used SVM with a polynomial kernel. For optimizing the parameters a “grid-search” was performed on *C* and *d* parameters: the exponential growth of *C* = 2*^N^* was tested, with *N* varying from −1 to 15 and the polynomial degree (*d*) was tested with the following values {1, 2, 3, 4, 5}. In study 3.2, we used a OCSVM and a GMM. Concerning the SVM, we used the RBF kernel and for optimizing the parameters a “grid-search” was performed on *γ* and *n* parameters: with *γ* varying from 1 × 10^−6^ to 1 × 10^−4^, for wLBP, and *γ* varying from 1 × 10^−3^ to 5 × 10^−2^, for GLCM; and the *n* was tested from 1 × 10^−2^ to 2 × 10^−2^. The optimization of the parameters was performed by a nested validation procedure, therefore the evaluation is done in the test set which was not used for that purpose. More details will be given in the following sections. Concerning the GMM, the number of components varied from 2^3^ to 2^7^ and the covariance matrix was not conditioned to be diagonal. For the OCSVM we used an implementation available in the Matlab-based (Mathworks, version R2012b) toolbox “LibSVM” [46]) and concerning the GMM, we used the Matlab-based (Mathworks, version R2012b) toolbox “Netlab” [47].

### 4.5. Study 1: Impact of Fingerprint Segmentation

As in the traditional approaches, the classification is performed for each material individually. Therefore, the fake samples used for training and testing are made of the same type of material. The live samples were divided in equal parts according to the number of materials and each part was used combined with the spoof samples of each material. In this study we consider the results obtained with and without segmenting the images. Also, we present the results obtained for each material and the median result of all materials in order to compare it with some state-of-the-art results. In this study, the classification error was obtained by averaging the misclassification error rate in 50 runs and, in each run, the data was divided randomly in 62.5% of the samples for training and 37.5% for testing. The training test was by its turn divided in the same way and the optimization of the parameters was performed by nested validation in this dataset.

### 4.6. Studies 2 and 3: From Supervised Classification to Semi-Supervised Classification

As already stated before, we consider the segmentation step to be crucial to the fingerprint liveness detection problem as it is in the fingerprint recognition field. Examples were showed that illustrate well the differences observed in the background areas in fingerprint images from distinct datasets. Naturally this factor will impact the liveness detection performance considering that the background will a have similar nature and on the other hand, ideally for the liveness detector, a live sample will differ from a spoof one. The fact that in some fingerprint images the background may constitute nine tenths of the image, which will surely impact on the liveness detection method. Taking account of these observations, we restrict the following study to the segmented images.

#### 4.6.1. Study 2

In the “mixed sets” study, the classification is performed within a set composed by a mix of samples made by all types of materials. Considering that, in practice, the system is not aware *a priori* what is the kind of fake sample to be used in an attempt to fool the system we note that the classification performed in study 1 is not very realistic. The study 2 consists in applying the same methodology used within each material in study 1 but now in a set comprising all the types of materials. In this study, we assume that we have information about all the possible materials (since the training and test sets comprise all the different materials used for making the fake samples) but we do not know which one will be selected by the intruder. In this study the classification error was obtained by averaging the misclassification error rate in 50 runs and in each run the data was divided randomly in 62.5% of the samples for training and 37.5% for testing. The training test was by its turn divided in the same way and the optimization of the parameters was performed by nested validation in this dataset.

#### 4.6.2. Study 3

In the third study we aim to go further and assume that there is no complete knowledge about the kind of material will be used in a spoof attack to be performed. In this specific situation, this means that the system is not aware of all possible types of materials used to build the fake samples or none at all. Two different situations will be consider: in study 3.1 we use one material to test and the remaining materials to train; in study 3.2 we do not assume any knowledge about the fake samples, we use only the real samples train our model and perform a “one class” classification study.

##### Study 3.1

In this approach, the “leave one material out” study, we assume that we know some possible materials but we do not have knowledge about the one used in the spoofing attack that is going to be perpetrated. So, to perform the classification we use one material to test and the remaining materials to train. We used a *k*-fold cross-validation in which, for *k* sets of materials, 1 was used for testing and k − 1 were used for training. The classification error was the mean of the *k* misclassification rate (one for each fold). Also, in this last study, we used the optimization of parameters as described previously. The real samples are divided in *k* equal parts and each one is combined with one dataset of fake samples.

##### Study 3.2

Then we moved a step further aiming to perform the semi-supervised approach to our problem. We intended to extend the lack of knowledge about the fake samples to its limit. Therefore, we use for training only the real/live samples and then we perform the testing step on a set composed proportionally by real/live and fake/spoof samples (the latter of only one specific type of material at each time). In this study, for *k* sets of materials, we consider *k* folds in which 1 material was used for testing and the classification error was the mean of the *k* misclassification rate (one for each fold). The training step is done using only the information of the real samples. Considering that the number of features for wLBP was considerably high, a PCA feature selection method was applied using the training data. Then we trained and tested our model using only the best features chosen. Since the set of characteristics to retain is optimized, the set of features and its cardinality is variable according to the datasets we studied. Experiments were run using two classifiers: a OCSVM and a GMM. Concerning the OCSVM, we chose the RBF kernel (which showed to perform better than the linear or the polynomial). For optimizing the parameters a “grid-search” was performed on *γ* and *n* parameters as described previously. Concerning the GMM, the number of components varied from 2^3^ to 2^7^ and we tested the two types of covariance, the one that obeys the condition that the covariance matrix has to be diagonal and the other that do not impose that condition, obtaining better results for the second one, which are the results presented.

### 4.7. Performance Evaluation

#### 4.7.1. Evaluation Metrics

The metrics we used are the ones suggested to evaluate the performance of presentation attack detection algorithms by *ISO/IEC 30107-3 Presentation Attack Detection* [48]. This standardization project focuses on techniques for the automated detection of presentation attacks undertaken by data capture subjects at the point of presentation and collection of the relevant biometric characteristics.

The “Normal Presentation Classification Error Rate” (NPCER) is given by the proportion of normal (live) presentations incorrectly classified as attack (non-live) presentations and the “Attack Presentation Classification Error Rate” (APCER) is given by the proportion of attack (non-live) presentations incorrectly classified as normal (live) presentations. The *Average Classification Error Rate (ACER)* is given by the mean of the NPCER and the APCER error rates. The ACER is the metric used to evaluate the results and corresponds to the misclassification rate of the classifier.

(1) $$\text{ACER}=\frac{\left(\text{NPCER}+\text{APCER}\right)}{2}$$

#### 4.7.2. Time Efficiency

Since the methods were implemented in MATLAB without any efficiency concerns, a straightforward assessment of the time efficiency is not fair. Nevertheless, some comments on the running time are in order. The most demanding operation is clearly the feature extraction, being the time duration of the remaining operations negligible when using the anti-spoofing system. The feature calculation time, especially concerning the wLBP method, grows with the size of the images. We followed the option of the authors of the method [36], normalizing the iris images into the same size (400 × 400). For the GLCM method, the feature extraction is significantly faster than for the wLBP method so we kept the original size of the images (that could already have been reduced in the case of segmented images). While the GLCM does not raises any concern for real-time applications, the wLBP extraction took several seconds per image in our implementation. However, as shown by [49], LBP implementation can be brought much closer to real time implementations. Concerning the SIFT part, an option includes replacing SIFT by a fast approximation (such as SURF) to weight the LBP.

## 5. Results and Discussion

### 5.1. Discussion of Results Obtained in Study 1

The two feature extraction methods were first applied testing the different materials separately (study 1), like presented in Section 4.5. Tables 2, 3, 4 and 5 present the results obtained for the Biometrika, Crossmatch, Italdata and Swipe subsets, respectively, using the whole image and the segmented fingerprints.

For the Biometrika dataset, the best result is obtained for the Ecoflex material for both wLBP and GLCM methods, and also for both non-segmented and segmented images, respectively 1.48% and 0.39%; 1.22% and 1.48%; see Table 2.

For the Crossmatch dataset, the best result, for wLBP, for non-segmented images, 1.93%, is obtained for the Play-Doh material, and for segmented images, 0.09%, is obtained for the Latex material; and for the GLCM method, for non-segmented images,1.64%, is obtained for the Wood Glue material, and for segmented images, 2.35%, is obtained for the Body Double material; see Table 3.

For the Italdata dataset, the best result, for wLBP, for non-segmented images, 2.29%, is obtained for the Modasil material, and for segmented images, 1.92%, is obtained for the Ecoflex material; and for the GLCM method, for non-segmented images, 1.21%, is obtained for the Ecoflex material, and for segmented images, 1.35%, is obtained for the Ecoflex material; see Table 4.

For the Swipe dataset, the best result, for wLBP, for non-segmented images, 6.77%, is obtained for the Play-Doh material, and for segmented images, 7.10%, is obtained for the Body Double material; and for the GLCM method, for non-segmented images, 5.87%, is obtained for the Play-Doh material and for segmented images, 6.94%, is obtained for the Body Double material; see Table 5.

Regarding the type of material we can notice that, for both methods, the material that leads to the best error rate is different for each of the four sensors. Nevertheless, we should notice that not all materials are used for all sensors. However, for the sensors that use the same materials this observation holds: for Biometrika and Italdata the best result corresponds to different materials however the Ecoflex predominates, and for the Crossmatch and Swipe there are no material that seems to lead to better results than all the others.

Comparing the results of non-segmented and segmented images, we observe that for wLBP the best result improve with the segmentation (with only one exception) however individually for each material the results with the segmentation does not always improve. Regarding the GLCM, we observe that even the best result does not always improve. Nevertheless, we sustain the necessity of performing the segmentation step with the fact that the background of the images vary among the different types and we believe that the feature extraction is highly biased by the background when we use the whole image. The discrepancies occur due to the fact that the descriptors are not equally sensitive to features observed.

In an overall analysis, the best result for wLBP without segmentation, 1.48%, is obtained for the Ecoflex material regarding the Biometrika sensor; and with segmentation, 0.09%, is obtained for the Latex material regarding the CrossMatch sensor. In an overall analysis, the best result for GLCM, without segmentation, 1.21%, is obtained for the Ecoflex material regarding the Italdata sensor; and with segmentation, 1.35%, is obtained for the Ecoflex material regarding the Italdata sensor.

In Table 6, a comparison between types of fake fingerprint samples is presented, where it can be observed which combination of mold and sensor results in higher misclassification rate. We can thereby observe that, concerning the wLBP method, the Play-Doh material may be considered “more difficult” to detect than the others and concerning GLCM is the Wood Glue material that presents a higher error rate.

### 5.2. Discussion of Results Obtained in Study 1 and from State-Of-The-Art Methods

In this section we discuss the results of some state-of-the-art methods that were evaluated in the same datasets used in our work. Therefore, we present in Table 7 the three best methods of the Fingerprint LivDet2013 Competition: UniNap1 [33], Anonym2 [33] and Dermalog [33]; and other four state-of-the-art methods evaluated in the LivDet2013 dataset AugLBP [13]; AugCN [13]; HIG_best [25] and Pore Analysis [24]. We only present the average errors for each dataset because the results are not available by material for the other methods. The results were all rounded to meet the minimum precision of the results presented in the literature.

It has to be stressed that the evaluation protocol in LivDet2013 and in the other works are different. In the competition, the algorithm was trained with one training set and then evaluated with the test set. In the other methods the protocols used for the classification step are variable. Nevertheless, is still pertinent to compare the results in order to show that the feature extraction methods used in the present work are not out of the range of the state-of-the-art results evaluated in the same dataset.

From Section 5.1 we can calculate the values for ACER obtained in study 1, using segmented images, for wLBP 2.0%, 0.1%, 3.2% and 9.5%, respectively for Biometrika, Crossmatch, Italdata and Swipe and the average of the four datasets is 3.7% with a standard deviation of 4.1%; for GLCM 7.7%, 5.3%, 4.0% and 7.6%, respectively for Biometrika, Crossmatch, Italdata and Swipe and the average of the four datasets is 6.2% with a standard deviation of 1.8%.

Observing the latter values and Table 7, it can be concluded that the results obtained in our experiments may not outperform the other results in all datasets but in average they are positioned second and third best. In the case of the Crossmatch dataset, the results achieved by both our tested feature extraction methods outperform considerably the others. This may be due to the fact that we segmented the images and, although we do not have information about all the methods, we believe that several of the methods do not perform the segmentation. We recall that in this dataset the images are significantly dominated by the background area. The only exception is the method based in convolutional networks [13] which has lower mean error rate than the presented GLCM method, however we note the fact that this method determine a region of interest before extracting the liveness detection features.

### 5.3. Discussion of Results Obtained in Study 2

Considering that, in practice, the system is not aware *a priori* what is the kind of fake sample to be used in an attempt to fool the system the classification performed in study 1 is not very realistic. Then different studies were applied aiming to overcome this limitation, presented in Section 4.6.1. In study 2 it is assumed that we have information about all the possible materials used in spoofing attacks to be performed since the training and test sets comprise all the different materials used for fake samples. The results presented in Table 8 show that there is not a significant discrepancy from the results of study 2. We can observe some differences in specific datasets but if we look to the average error rate the difference is almost unnoticeable.

### 5.4. Discussion of Results Obtained in Studies 3.1 and 3.2

Our aim to broad the classification approach in order to perform a semi-supervised classification instead of a supervised classification conducted to the study 3, presented in Section 4.6.2, in which we limit the knowledge of fake/spoof samples that is used in the training step. In a first attempt, we still use some sets of fake/spoof samples in the training set but the novelty is that in the training set we include a different type of fake/spoof samples, this is the denominated study 3.1. In study 3.2, we then perform a “full” semi-supervised classification by performing the training of our model using only the real/live samples and then using fake/spoof samples in the test step.

In Tables 9, 10, 11 and 12 we present the results of studies 3.1 and 3.2 (for the OCSVM and the GMM). These are the minimum ACER obtained using PCA for feature selection, the number of features were optimized for all subsets and differ among them. When using the RBF kernel for the SVM, the parameters *γ* and *n*, were optimized like described in Section 4.6.2 and the optimal parameters are different for each material as we are testing with only one material at a time and ignoring the other types of materials. Also, for the GMM the number of components was optimized and again the optimal value vary according to each subset.

Observing the results of Tables 9, 10, 11 and 12, we may conclude that, in terms of average results, for wLBP, the GMM approach outperforms for only one sensor (Crossmatch), but for GLCM, the GMM approach outperforms for all sensors. These results show that if there is no knowledge about the material to be presented in a spoofing attack then the one-class classifier approach is more suitable than the binary classifier approach used in study 3.1, where the model is trained with several materials but then a new material is encountered in the test phase. One important aspect is how much we are being optimistic when evaluating our models. A capable intruder will try to develop new materials for spoofing different from the known ones. So, even though these results are worse than the results obtained in studies 1 and 2, we consider the semi-supervised approach more realistic than the supervised one where the same material appears in the train and test phase assuming that the system developer will know all the spoofing materials.

## 6. Conclusions and Future Work

In this study we applied different classification studies in fingerprint liveness detection in order to broad the traditional approaches that use the knowledge about the fake/spoof samples for training the models. Therefore, in a first approach we mixed the fake materials in train and test sets instead of training and testing with only one specific material. However, this approach is still not very realistic since we assumed to have knowledge about all the possible spoof attacks. So, the next approach was to test with one material and train with the rest of the materials. As expected, this approach lead to worse results since we are using a complete unknown material in the test step. Finally, the last approach, which we consider the most worth following, consisted on using only the information of the real samples when training our model and then test it with real and fake samples. In fact, what we are performing is a semi-supervised classification characterizing the real samples and expecting our model to classify correctly as fake the spoof samples in the test set. Two different methods were used, a one-class SVM and a mixture of gaussians, being the best results produced by this latter one. Although the results of the semi-supervised approach are worse than the supervised classification, we still consider the first to be more realistic. In our opinion, is more adequate to evaluate the robustness of a liveness method to unknown spoof attacks not assuming complete or even partial knowledge about the fake/spoof samples to be used by an intruder. We consider the results obtained in our experiments encouraging to pursue this approach in future works broadening the study to other databases and also other biometric traits. This approach has raised interest recently in the liveness detection field as some referred works show, but, to the best of our knowledge has not been yet fully studied and in our opinion can be further explored. In this work, we also illustrate the variability in the background of fingerprint images among the different datasets used and we argue about the necessity of a segmentation step.

## Figures and Tables

**Table 1:** Some characteristics of the images from the LivDet2013 datasets.

**Dataset**	**Sensors**	**Images Resolution (dpi)**	**Images Sizes**	**Gray Scale Levels**
#1	Biometrika	569	315 × 372	256
#2	Italdata	500	640 × 480	256
#3	Crossmatch	500	800 × 750	256
#4	Swipe	96	208 × 1500	256

**Table 2:** Average Classification Error Rate (ACER) for each material of the Biometrika dataset, study 1 (ACER in %).

**Methods**	**Biometrika**									
	**Ecoflex**		**Gelatin**		**Latex**		**Modasil**		**Wood Glue**	
	***μ***	***σ***	***μ***	***σ***	***μ***	***σ***	***μ***	***σ***	***μ***	***σ***
wLBP	**1.48**	0.69	8.13	1.41	3.04	0.99	2.43	0.77	2.66	0.91
GLCM	**1.22**	0.59	4.39	1.46	6.63	1.47	7.86	1.54	10.21	2.35
wLBP (segmented images)	**0.39**	0.30	4.25	1.22	1.85	0.72	1.63	0.71	1.90	0.79
GLCM (segmented images)	**1.48**	0.76	4.90	1.29	9.97	1.75	6.66	1.47	15.63	2.52

**Table 3:** ACER for each material of the Crossmatch dataset, study 1 (ACER in %).

**Methods**	**Crossmatch**							
	**Latex**		**Wood Glue**		**Body Double**		**Play-Doh**	
	**Mean**	***σ***	**Mean**	***σ***	**Mean**	***σ***	**Mean**	***σ***
wLBP	16.79	1.056	16.71	0.81	16.84	0.74	**1.93**	0.48
GLCM	3.99	0.65	**1.64**	0.45	1.74	0.36	4.72	0.93
wLBP (segmented images)	**0.09**	0.12	0.11	0.12	0.11	0.12	0.10	0.12
GLCM (segmented images)	7.02	1.78	3.30	1.09	**2.35**	0.67	8.60	1.86

**Table 4:** ACER for each material of the Italdata dataset, study 1 (ACER in %).

**Methods**	**Italdata**									
	**Ecoflex**		**Gelatin**		**Latex**		**Modasil**		**Wood Glue**	
	**Mean**	***σ***	**Mean**	***σ***	**Mean**	***σ***	**Mean**	***σ***	**Mean**	***σ***
wLBP	2.47	0.92	3.47	1.46	3.05	1.09	**2.29**	0.92	3.86	1.03
GLCM	**1.21**	0.52	6.26	1.56	7.15	1.71	3.30	1.48	6.97	1.38
wLBP (segmented images)	**1.92**	0.65	4.69	1.13	3.22	0.93	2.36	0.90	3.67	1.12
GLCM (segmented images)	**1.35**	0.80	4.64	1.05	4.73	1.24	2.65	1.14	6.44	1.65

**Table 5:** ACER for each material of the Swipe dataset, study 1 (ACER in %).

**Methods**	**Swipe**							
	**Latex**		**Wood Glue**		**Body Double**		**Play-Doh**	
	***μ***	***σ***	***μ***	***σ***	***μ***	***σ***	***μ***	***σ***
wLBP	13.49	1.64	6.85	1.01	9.83	1.25	**6.77**	1.26
GLCM	8.71	1.87	6.06	1.14	7.46	1.71	**5.87**	1.32
wLBP (segmented images)	10.92	1.61	8.65	1.42	**7.10**	1.38	11.43	1.52
GLCM (segmented images)	8.62	1.70	7.54	1.55	**6.94**	1.54	7.43	1.17

**Table 6:** ACER for the each type of fake mold and the respective sensor (ACER in %).

**Material**	**wLBP**	**Dataset**	**GLCM**	**Dataset**
Ecoflex	1.92	Italdata	1.48	Biometrika
Gelatin	4.69	Italdata	4.90	Biometrika
Latex	10.92	Swipe	9.97	Biometrika
Modasil	2.36	Italdata	6.66	Biometrika
Wood Glue	8.65	Swipe	**15.63**	Biometrika
Body Double	7.10	Swipe	6.94	Swipe
Play-Doh	**11.43**	Swipe	7.43	Swipe

**Table 7:** Results of state-of-the-art methods (ACER and average in %).

**Methods**	**State-Of-The-Art Methods**					

	**Biometrika**	**Crossmatch**	**Italdata**	**Swipe**	**Average**	

	***μ***	***μ***	***μ***	***μ***	***μ***	***σ***
UniNap1 [33]	4.7	31.2	3.5	13.8	13.3	12.8
Anonym2 [33]	1.8	49.4	0.6	6.1	14.5	23.4
Dermalog [33]	1.7	49.9	0.8	3.5	14.0	24.0
Aug LPB [13]	1.7	49.5	**2.3**	**3.3**	14.2	23.5
Aug CN [13]	**0.8**	**3.3**	2.5	7.7	**3.6**	2.9
HIG [25]	3.9	28.8	1.7	14.4	12.2	12.7
Pore Analysis [24]	2.2	34.9	1.0	-	12.7	19.2

**Table 8:** ACER for Each Dataset and Their Average, Study 2 (ACER and average in %).

**Methods**	**Study 2**									

	**Biometrika**		**Crossmatch**		**Italdata**		**Swipe**		**Average**	

	***μ***	***σ***	***μ***	***σ***	***μ***	***σ***	***μ***	***σ***	***μ***	***σ***
wLBP (segmented images)	1.56	0.30	**0.09**	0.05	2.18	0.52	9.22	0.81	3.26	4.07
GLCM (segmented images)	7.94	0.69	5.94	0.66	**2.88**	0.42	8.14	0.81	6.23	2.44

**Table 9:** ACER for the Biometrika dataset, study 3 (ACER in %).

**Methods**		**Biometrika**						
		**Ecoflex**	**Gelatin**	**Latex**	**Modasil**	**Wood Glue**	***μ***	***σ***
Study 3.1	wLBP	4.63	6.25	**1.63**	3.88	3.63	**4.00**	1.68
	GLCM	**19.63**	40.25	48.75	30.75	47.13	37.30	12.16
OCSVM	wLBP	**9.50**	20.88	17.00	11.75	17.25	15.28	4.58
	GLCM	**15.88**	18.13	43.50	28.75	48.00	30.85	14.53
GMM	wLBP	**7.75**	19.00	18.00	12.13	16.25	14.63	4.66
	GLCM	**10.88**	11.88	28.75	20.13	33.63	21.05	10.07

**Table 10:** ACER for the CrossMatch dataset, study 3 (ACER in %).

**Methods**		**Crossmatch**					
		**Latex**	**WoodGlue**	**BodyDouble**	**Play-Doh**	***μ***	***σ***
Study 3.1	wLBP	8.53	0.08	**0.18**	2.31	2.78	3.79
	GLCM	55.64	43.11	27.56	**17.51**	35.96	16.83
OCSVM	wLBP	34.93	20.00	14.58	**11.02**	20.13	10.53
	GLCM	45.24	24.89	33.78	**23.02**	31.73	10.16
GMM	wLBP	2.22	**0.98**	1.33	1.24	**1.44**	0.54
	GLCM	47.82	**16.00**	24.89	17.69	**26.60**	14.66

**Table 11:** ACER for the Italdata dataset, study 3 (ACER in %).

**Methods**		**Italdata**						
		**Ecoflex**	**Gelatin**	**Latex**	**Modasil**	**Wood Glue**	***μ***	***σ***
Study 3.1	wLBP	0.88	4.50	3.13	7.50	13.63	**5.93**	4.93
	GLCM	37.25	**32.63**	49.63	42.25	44.25	41.20	6.53
OCSVM	wLBP	**16.88**	26.00	25.88	23.00	30.63	24.48	5.05
	GLCM	**33.88**	39.38	35.13	37.00	43.50	37.78	3.81
GMM	wLBP	**11.25**	16.50	17.25	17.63	21.00	16.73	3.51
	GLCM	10.63	**10.25**	13.00	10.75	20.13	**12.95**	4.16

**Table 12:** ACER for the Swipe dataset, study 3 (ACER in %).

**Methods**		**Swipe**					
		**Latex**	**WoodGlue**	**BodyDouble**	**Play-Doh**	***μ***	***σ***
Study 3.1	wLBP	**15.28**	17.34	16.19	33.98	**20.70**	8.90
	GLCM	54.16	54.13	49.31	**33.43**	47.76	9.82
OCSVM	wLBP	44.24	46.19	46.57	**33.70**	42.68	6.07
	GLCM	45.89	44.54	37.24	**35.38**	40.76	5.23
GMM	wLBP	37.29	48.76	43.37	**26.28**	38.93	9.64
	GLCM	36.14	33.76	20.86	**20.52**	**27.82**	8.29
